# The “Spinner” Illusion: More Dots, More Speed?

**DOI:** 10.1177/2041669517707972

**Published:** 2017-05-07

**Authors:** Hiroshi Ashida, Alan Ho, Akiyoshi Kitaoka, Stuart Anstis

**Affiliations:** Kyoto University, Kyoto, Japan; Ambrose University, Alberta, Canada; Ritsumeikan University, Kyoto, Japan; University of California, San Diego, CA, USA

**Keywords:** psychophysics, speed perception, visual illusion, visual motion

## Abstract

The perceived speed of a ring of equally spaced dots moving around a circular path appears faster as the number of dots increases (Ho & Anstis, 2013, Best Illusion of the Year contest). We measured this “spinner” effect with radial sinusoidal gratings, using a 2AFC procedure where participants selected the faster one between two briefly presented gratings of different spatial frequencies (SFs) rotating at various angular speeds. Compared with the reference stimulus with 4 c/rev (0.64 c/rad), participants consistently overestimated the angular speed for test stimuli of higher radial SFs but underestimated that for a test stimulus of lower radial SFs. The spinner effect increased in magnitude but saturated rapidly as the test radial SF increased. Similar effects were observed with translating linear sinusoidal gratings of different SFs. Our results support the idea that human speed perception is biased by temporal frequency, which physically goes up as SF increases when the speed is held constant. Hence, the more dots or lines, the greater the perceived speed when they are moving coherently in a defined area.

## Introduction

Visual motion provides us with vital information about the environment necessary for our daily survival. The ability to perceive the speed and direction of external moving objects enables us to act on the objects or to navigate through the environment safely without being harmed. While detection of motion and identification of direction have been studied extensively by psychophysicists, physiologists, and computational neuroscientists, our knowledge of speed perception is rather limited in terms of its phenomenology and underlying mechanisms (e.g., see a review by Nishida, 2011). One possible factor limiting our understanding of speed perception and vision in general is the inverse projection problem (Palmer, 1999). This problem arises because an infinite number of distal environmental objects with different shapes and sizes seen in the three-dimensional (3D) world can cast the same two-dimensional (2D) optical image onto the retina, introducing ambiguities difficult to solve. A moving distal environmental object further extends the inverse projection problem into the time domain and possibly introduces more uncertainty and inaccuracy for the visual system in processing visual motion information. Since the claims that the human visual system analyses complex visual images by their spatial frequency (SF) content like a Fourier analyzer (Campbell & Robson, 1968; Sachs, Nachmias, & Robson, 1971), researchers have been using sinusoidal gratings extensively to study human vision. The speed of a moving stimulus used in researches is consequently expressed in terms of its spatial and temporal frequencies, and is susceptible to biases from other stimulus properties such as its luminance contrast (Thompson, Brooks, & Hammett, 2006; Thompson, 1982) and colour (Cavanagh, Tyler, & Favreau, 1984).

**Figure 1.**
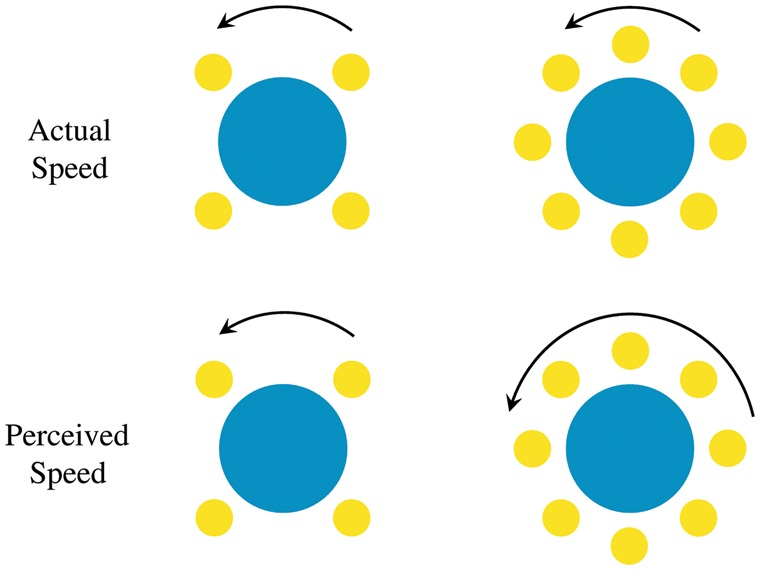
The spinner illusion. The four and eight yellow dots revolve around the blue disks at the same physical speed (top), but the perceived speed appears to be faster with eight dots than with four dots (bottom). Arrow lengths symbolize speeds. The blue disks were not included in the original illusion work, but are shown here for illustration purposes.

A counterintuitive bias in speed perception was recently demonstrated by Ho and Anstis (2013) in the 2013 Best Illusion of the Year contest. They later redesigned it as “the spinner illusion” using simpler disk elements, as shown in Figure 1 (see also Appendix Movie 1). The four yellow dots on the left and the eight yellow dots on the right were both set to revolve at the same rate, yet all observers consistently reported seeing the dots on the right as rotating faster than those on the left. In the demonstration video, the number of dots on the right increases from 4 to 8 and then 12, with the rotation seeming faster with each increase.

The spinner illusion is interesting because there is no obvious reason in physics why increasing the number of dots should increase their apparent speed, assuming that all the dots are clearly visible. Ho and Anstis (2013) originally suggested that the greater retinal blur caused by more dots might make them look faster. While it is known that motion streaks can enhance motion perception (Apthorp et al., 2013; Geisler, 1999), their influence on speed perception has not been established. We therefore measured the spinner effect with sine wave grating stimuli that are smooth and less susceptible to smearing.

An alternative account would treat this illusion as a partial failure to compute speed from the temporal frequency (TF) and SF of the stimulus. While local speed is obtained by local TF divided by SF in theory, this computation might be performed only in an approximate way. It has been reported that perceived speed of translating one-dimensional (1D) sinusoidal gratings depends on their SF. Campbell and Maffei (1981) studied rotating 1D gratings and found that their perceived speed followed an inverted-U function of spatial frequency. Diener, Wist, Dichgans, and Brandt (1976) reported increased perceived speed at higher SFs measured by magnitude estimation, a finding that was later echoed by Chen, Bedell, and Frishman (1998) using a 2AFC procedure. Smith and Edger (1990) showed opposite results that both perceived speed and TF decreased as the SF increased, but they pointed out that the results may depend on the range of SFs used.

In the spinner illusion (Figure 1), SF along the circular path of motion increases as the number, and thus density, of dots increases. Possibly this illusion directly reflects the spatiotemporal characteristics of the visual system as described earlier. If so, one extreme possibility would be that speed judgments of rotating stimuli are made on the basis of local TF, ignoring the SF. Or they might be made as a compromise between the speed and TF of a rotating stimulus. The results in the literature are not conclusive. One problem is that it is not straightforward to apply results from the linear gratings to the configuration of a rotating spinner illusion. We first need to confirm that simple sinusoidal patterns give similar illusions, and then to examine the spatiotemporal characteristics of the illusion parametrically.

**Figure 2.**
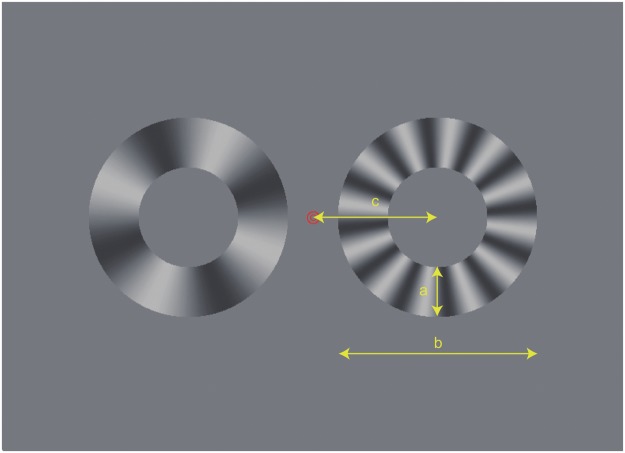
The basic stimulus configuration. Left: matching stimulus of 4 c/rev, Right: test stimulus of 12 c/rev as an example. Both stimuli rotated in the same direction. The test and matching sides as well as their yoked direction of rotation were randomized and balanced across trials. In Experiment 1: a = 2.2°, b = 8.8°, and c = 5.5°. In Experiments 2 and 3: a = 2.75°, b = 11.0°, and c = 6.9°.

We therefore investigated the effect of SF on the spinner illusion in radial gratings (Figure 2). First, we confirmed that the spinner illusion occurs with simple radial sinusoidal gratings for naïve participants (Experiment 1). Sinusoidal modulation is also effective in minimizing the effect of blur, as image blur does not yield motion streaks but only reduces the effective contrast. Second, we examined whether the illusion depends more on speed itself or TF, by taking more detailed measurements from trained participants (Experiment 2). Finally, we confirmed that a similar illusion occurs for translational motion of one-dimensional gratings (Experiment 3). We will then discuss the cause of this illusion in terms of general spatiotemporal integration.

## Experiment 1

The point of subjective equality (PSE) in perceived speed for two simultaneously presented rotating radial sinusoidal gratings of different radial spatial frequencies (RSFs) was assessed psychophysically using a method of constant stimuli. Specifically, the rotating speed of the matching stimulus (RSF of 4 c/rev: cycle per revolution^1^) was varied randomly between trials and was compared with that of the test stimulus (RSF of either 8, 10, or 12 c/rev; used uniquely in separate experimental blocks) that was kept constant at 0.33 rev/s, in order to obtain psychometric functions for the estimation of their PSEs. We expected the resulting PSE to be higher than the veridical speed of the test stimuli (0.33 rev/s) if the spinner effect occurred.

### Methods

#### Participants

Eight psychology undergraduate students (6 females and 2 males, aged 19–22) in Kyoto University participated to fulfill partial requirement of a course. They were naïve as to the specific purpose of the experiment, although they had seen a demonstration of the spinner illusion prior to the experiment. All had normal or corrected-to-normal vision.

#### Apparatus and stimuli

Stimuli were generated and presented by using PsychToolBox 3 (Brainard, 1997; Pelli, 1997) on MATLAB (The Mathworks, Inc., Natick, MA, USA). They were presented on one of the three monitors: two 24-inch (BenQ XL2411) and a 23-inch (Mitsubishi RDT233WX) LCD with 1920 × 1080 resolution and 60 Hz refresh rate, driven by PCs running Microsoft Windows 7. Participants rested their heads comfortably on a chin rest during experiment with a viewing distance at 50 cm. The mean luminance was 40 cd/m^2^ for one BenQ and 100 cd/m^2^ for the others. The luminance profile of each screen was measured and was linearized using a photometer (Minolta LM1).

The basic stimulus configuration is shown in Figure 2 (see also Appendix Movie 2). Two radial gratings, each presented inside a ring-shaped window subtending a visual angle of 8.8°, were centered 5.5° laterally on each side of the central red fixation mark. The width of the rings was 2.2°. The two yoked stimuli always appeared and disappeared on the screen simultaneously. They had a sluggish temporal envelope: Their luminance contrast was increased linearly from 0% to 50% in 0.25 s and stayed at 50% for 0.5 s, and then decreased linearly back to 0% in 0.25 s. The two stimuli rotated in the same direction in each trial, and both directions were tested within each block of trials.

The RSF for the matching stimulus was 4 c/rev (0.64 c/rad), while the RSF of the test stimulus was picked from three preset values of 8, 12, or 16 c/rev (1.27, 1.91, and 2.55 c/rad) and stayed constant within each block of trials.

The speed for the test stimulus was fixed at 0.33 rev/s (2.09 rad/s) and the speed for the matching stimulus was picked randomly from seven preset values between 0.17 and 0.67 rev/s (1.05–4.19 rad/s).

### Procedure

The method of constant stimuli was used to present a yoked test-matching stimulus pair on each trial. The participants judged whether the radial grating inside the left or the right annulus window appeared faster in a 2AFC procedure and responded by pressing one of two designated computer keys.

Trials were organized in blocks by the three test RSFs. One block consisted of 28 trials (7 manipulated speeds × 2 sides × 2 rotation directions), and each block was repeated four times in a pseudorandom order, resulting in 16 measurements per manipulated matching speed. The computer programme randomized the manipulated speed variations, direction of stimulus-pair rotation, and the presentation sides of test-matching stimuli.

### Results and Discussion

**Figure 3.**
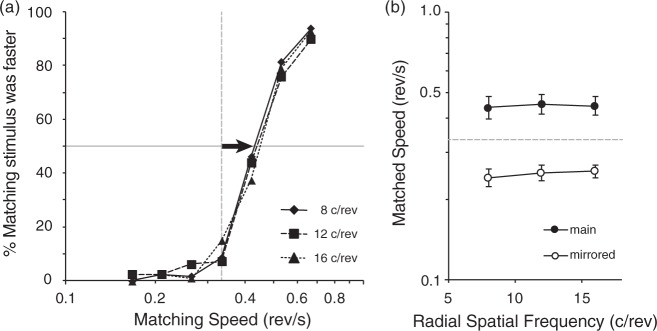
The results of experiment 1: (a) pooled psychometric functions from all the participants. The speed of the 4 c/rev matching stimuli needed to be increased from 0.33 rev/s to match the speed of 8 to 16 c/rev test stimuli (arrow). (b) The averaged PSE values (matched speeds) across participants, plotted as a function of RSF with 95% confidence intervals. The dashed straight line in each panel shows the actual speed of the test stimuli. (b) The filled symbols represent the main results, while the open symbols represent the auxiliary results with the speed of the higher SF stimuli manipulated; they are above and below the actual speed line in about the same distances in the log scale, so the two results agree quantitatively as to the amount of speed overestimation. The shifts of the psychometric functions (a) and thus the shifts of the matching speed (b) are all consistent with speed overestimation for higher RSFs.

Probit analysis (Finney, 1971; with the scripts by Johnson, Dahlgren, Siegfried, & Ellis, 2013) was used to estimate each participant’s PSE in perceived speed for each test RSF. The results were collapsed across presentation sides of stimuli and their rotation directions. Figure 3(a) shows the averaged psychometric functions from all participants, and Figure 3(b) shows estimated PSE values for all test-matching paired stimuli. It is evident from the data that the speed of the matching stimulus needed to be turned up beyond the test speed of 0.33 rev/s to match the perceived speeds of the test stimuli. In other words, the speed of a higher RSF test stimulus was consistently overestimated by the participants, which agreed with the original spinner illusion observation.

Specifically, the participants required the 4 c/rev matching stimulus to rotate 32% to 36% faster than the actual speed of the high-RSF test stimuli in order to match the perceived speeds. There was no significant difference in the matched speeds among the test stimuli of 8, 12, and 16 c/rev, *F*(2,14) = 0.334, *p* = 0.721 by repeated-measure ANOVA.

The speed overestimation of the test stimuli (>30%) here is similar to the report of 33% for 8 versus 16 dot condition of the original spinner illusion (Anstis & Ho, 2014). It is, however, not consistent that our data from Experiment 1 did not reveal differences among the three high-RSF tests. A possible factor might be compression of perceived speed in a higher RSF range; tiny differences for higher RSFs may be buried in noise under the ceiling effect.

We therefore ran an auxiliary experiment with a different speed manipulation in a mirrored fashion where the speeds of the higher RSF stimuli (8, 12, 16 c/rev) were manipulated to match the apparent speed of the 4 c/rev reference stimulus. The reference speed used here was the same as the test speed in the main experiment (0.33 rev/s = 2.09 rad/s). We used the same set of apparatus, but used PsychoPy (Peirce, 2007) for stimulus generation and experimental control. The manipulated speed for the test stimulus was picked randomly from seven preset values between 0.17 and 0.47 rev/s (1.05–2.96 rad/s). Nine different naïve participants (2 females and 7 male psychology students, aged 19–22) participated to fulfill partial requirement of a course. Their results are shown in Figure 3(b) by open symbols. Similar to the findings from the main experiment, no significant difference in the estimated PSE values was observed among the 8, 12, and 16 c/rev test stimuli, *F*(2,16) = 1.063, *p* = .369 by repeated-measure ANOVA. Participants required the speed of higher RSF test stimuli to be decreased to 72% to 76% of the actual 4 c/rev reference stimulus speed to perceptually match the perceived rotational speed of the yoked stimulus pair. By taking the reciprocals of these PSE values, the result corresponded to a speed overestimation of higher RSF stimuli by 31% to 39%, an observed spinner effect which is quantitatively consistent with the main results of 33% to 36% obtained in the main experiment. Thus, the results here mirrored those of the main experiment, indicating that the flat results among the test RSFs is not an artefact of the ceiling effect.

We then reasoned that the insignificant differences observed could possibly be due to a high level of internal noise from our naïve participants, lack of regression precision due to rough sampling of test speeds, or possible dependence of the effects upon the test speed. In Experiment 2, we therefore further assessed the spinner effect more extensively using trained participants with a staircase method while adopting a wider range of test RSFs in combination with multiple test speeds as manipulated variables.

## Experiment 2

### Participants

One of the authors and two psychophysically trained participants were tested (one female and two males, aged 22–47). The two participants other than the author were naïve as to the specific purpose of this experiment, and were paid for their time at the university standard rate. All participants had normal or corrected-to-normal vision.

### Apparatus and Stimuli

The stimuli were generated by using PsychoPy 1.82 (Peirce, 2007), running on an Apple MacBook Pro 15, and were presented on a 19-inch CRT (EIZO T761) with 1024 × 768 resolution and 75 Hz refresh rate. The screen was viewed from the distance of 45 cm with the aid of a chin rest. Luminance profile of the screen was measured and linearized with a photometer (Photo Research PR-655).

The basic stimulus configuration was the same as in Experiment 1 but 25% larger in size; the ring-shaped windows subtended 11°, with the annulus subtending 2.75° in width (Figure 2). These two stimulus windows were centered laterally at an eccentricity of 6.9° on both sides of the central fixation mark. The time course of stimulus presentation was the same, too: linear increase for 0.25 s, staying at 50% for 0.5 s, and linear decrease to zero for 0.25 s.

The RSF of the matching stimulus used here was again 4 c/rev (0.64 c/rad) as used in Experiment 1. Five test SFs were used: 2, 8, 12, 16, and 20 c/rev (0.32, 1.27, 1.91, 2.55, 3.18 c/rad) in combination with three test speeds: 0.17, 0.33, and 0.50 rev/s (1.05, 2.09, and 3.14 rad/s).

### Procedure

Participants were given the same 2AFC discrimination task as described in Experiment 1, judging the faster stimulus. All participants were tested with 15 different test stimulus conditions (i.e., the five test RSFs × three test speeds as stated earlier) to complete this experiment. A staircase method (1-up and 1-down) with smaller preset step sizes was used for capturing more precise changes in participants’ responses around the PSE for each test stimulus.

Each condition was tested in separate blocks of randomly interleaved double staircases (one staircase for one direction of motion). Each staircase was terminated after 28 trials (i.e., 56 trials per run with double staircases). Each block was repeated three times, bringing a grand total of 168 trials per stimulus condition.

### Results

Responses were pooled for each test stimulus condition within individual participant. The perceived matched speed (PSE) for each test stimulus condition was estimated with 95% confidence intervals using probit analysis (Finney, 1971; Johnson et al., 2013).

**Figure 4.**
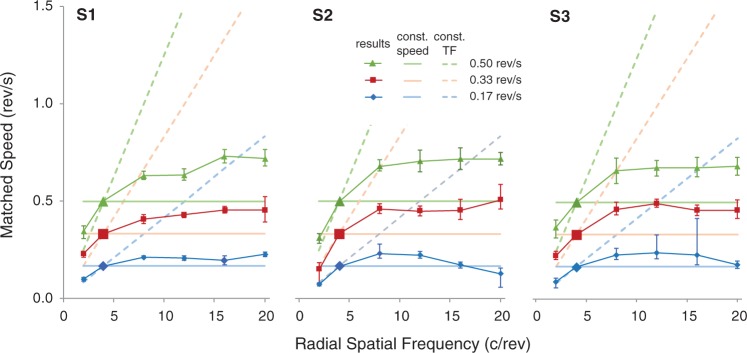
Results of Experiment 2 for all three participants in linear scales. PSE speeds are plotted as a function of the test RSF for each test speed. Error bars show 95% confidence intervals. The larger data symbols at 4 c/rev denote the matching stimulus itself where its own PSEs were not measured. Pale solid horizontal lines depict the physical speeds of the three test stimuli, and the pale dashed lines depict the physical speeds that give constant TFs (i.e., hypothetical lines of matched speeds if the perceived speeds of test stimuli were determined solely by the local TF characteristics.).

Figure 4 shows the results for all three participants. Matched speeds (PSEs) of the matching stimulus to the test stimuli are plotted as a function of RSF for each participant in separate panels. Since the 4 c/rev stimulus was used as the matching stimulus, we assumed the veridical speed to be its perceived speed for each test speed level (marked by larger symbols in Figure 4). The results look very similar across all three participants. Most of the test stimuli of 8 c/rev RSF or greater required the matching speed to be set above the corresponding actual test speed, demonstrating overestimated perceived speed of higher RSF test stimuli as found in Experiment 1. On the other hand, the matching speeds for the 2 c/rev test stimulus all fell below their veridical speed lines, revealing an underestimation of perceived speed for the lower RSF stimulus.

**Figure 5.**
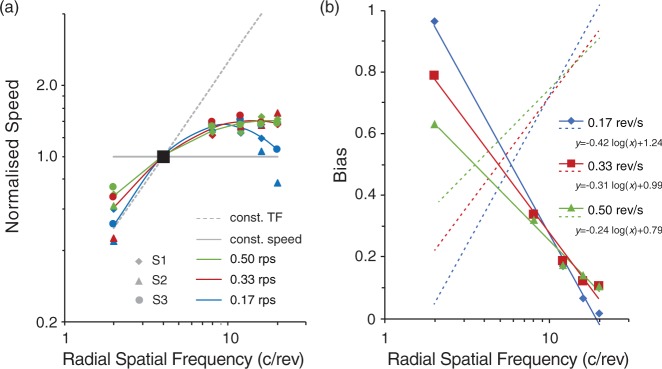
(a) The results of Experiment 2 normalized with the corresponding test speed in a log-log scale, with different symbols representing results of each participant (adapted from Figure 4). The large black square denotes the matching speed, which corresponds to the larger symbols in Figure 4. Solid curves show fitted double-log quadratic functions to the averaged data across participants for each test speed (*r*^2 ^> 0.99 for all). The 4 c/rev stimulus was used as the matching stimulus, and therefore no PSE estimate was made. The gray lines show equal speeds (solid) and equal-TFs (dashed). (b) Estimated TF bias for each test speed. Colored lines show fitted linear functions (*r*^2 ^> 0.98 for all) for TF weighting (*b*). Dotted lines represent the weights on actual speed (1 − *b*).

All the PSE curves fell within an area between the constant-TF and veridical test speed lines, and tended toward horizontal as the RSF of the test stimuli increased. These data reveal that the participants made a compromise between the actual speed and the TF of the moving stimuli in making their speed judgments on the revolving stimuli. The response functions are also very similar in trend across the three tested speed levels for all tested RSFs, with PSE values increasing as the RSF and the test speed increase. To compare the results across test speeds, each participant’s PSE data are normalized with the test speeds (i.e., divided by corresponding test speed) and replotted in a log-log scale as presented in Figure 5(a). Note that this normalized plot shows quadratic trends for all three test speeds; log-quadratic functions were fitted well to the averaged data across participants (*r*^2 ^> .99). The three curves nearly superimposed on each other except for the one obtained at the lowest test speed (0.17 rev/s), where PSEs for the lowest and highest RSFs were much lower than those for the faster test speeds. It is also noticed from the other two fitted curves that participants’ overestimation on perceived speeds for the higher RSF test stimuli started to level off at 8 c/rev. There are only subtle differences in speed overestimation among the stimuli of 8 to 16 c/rev, confirming our earlier finding in Experiment 1. We also noticed that the results from the three participants were quantitatively consistent except for data points at either end of RSFs examined.

Furthermore, from Figure 5(a), we see that perceived speed on the 2 c/rev stimulus lies much closer to the oblique TF line than the horizontal line, a characteristic that is opposite for the higher RSF stimuli. This means that speed perception is more heavily weighted by TF at low RSFs but more by the actual stimulus speed at high RSFs. To quantify such changes in weighing on speed perception by the TF characteristics of a revolving stimulus, we evaluated the contribution of TF by the following equation that can account for the matched speed as a weighted sum of the constant-TF speed and the stimulus’ actual angular speed:

$$m=b*S_{\mathrm{TF}}+(1-b)S$$

where *m* is the matched speed for a revolving stimulus, *b* is the TF-bias weighing factor, *S_TF_* is the constant-TF speed at each spatial frequency, and *S* is the veridical stimulus speed. TF would have no effect with *b* = 0 and the strongest effect with *b* = 1. Figure 5(b) shows *b* (solid lines) and 1 − *b* (dotted lines) as a function of the RSF, which were obtained by solving this equation for the averaged results in Figure 5(a) as *m* for each test speed. This plot reveals that the TF weighting on speed perception falls almost linearly in a log scale as the RSF of a stimulus increases. Even though the speed overestimation effect increases for stimuli of higher RSFs, the effect of TF on speed perception is actually stronger for stimuli of lower RSFs. This plot also indicates that the effect of TF is larger for a slower speed at the lowest RSF tested; the reason is not clear, but it could be an artefact of showing very thick stripes in narrow rings.

The curves may be subject to change by the choice of the matching stimulus. An auxiliary experiment performed by S1, however, suggested that such a nonlinear effect, if any, might not be substantial at least for our stimulus set; the speed of the 8 c/rev stimulus was matched to that of the 16 c/rev stimulus by the same procedure, and the speed of 16 c/rev stimulus was overestimated by 9.2% with the 95% confidence interval from 5.9% to 12.5%. The data from S1 in Figure 4 increased by 11.5% from 8 c/rev to 16 c/rev, which falls within this confidence interval.

In short, the spinner effect is observed with radial sinusoidal stimuli. Perceived speed of a test stimulus does increase with the RSF of the stimulus, weighted concomitantly by an increasing TF. But the magnitude of the spinner effect reaches a plateau relatively quickly at higher RSFs, indicating that the bias toward TF is actually smaller for higher RSFs. Therefore, possible difference among 8 to 16 c/rev stimuli could be easily buried in noise in Experiment 1 with naïve participants and with coarser grain of test speeds.

## Experiment 3

If the spinner illusion reflects a genuine SF dependent speed overestimation, it may not be specific to rotational motion. We can actually experience an analogous speed illusion in the demonstration movie with translating linear gratings (Appendix Movie 3), as also predicted from previous reports (Chen et al., 1998; Diener et al., 1976). In Experiment 3, we therefore assessed if analogous characteristics of the spinner illusion would be found in drifting vertical sine wave gratings. The SFs of the 1D gratings used here were roughly matched to the SF of the radial stimuli in Experiment 2.

### Methods

#### Participants

The same three participants as in Experiment 2 were tested.

#### Apparatus and stimuli

The apparatus was the same as in Experiment 2, also controlled with PsychoPy 1.82 (Peirce, 2007). The layout of the paired matching-test stimulus was similar to that used in Experiment 2. Here, two 1D vertical sinusoidal grating of different SFs, always drifting in the same direction, were presented inside two separate, 11° diameter circular windows on both sides of the central fixation point. Their maximum luminance contrast was 50%, presented to participants using the same trapezoidal time envelope as before.

The SFs were approximately matched to those of the radial gratings as follows; luminances were modulated horizontally in the frequencies of 2, 4, 8, 12, and 16 cycles per the circumference of the middle point of the ring in Experiment 2 (i.e., along the circle of 8.25° diameter), resulting in 0.08, 0.15, 0.31, 0.46, and 0.62 c/deg, respectively. The SF of the matching stimulus was 0.15 c/deg, and that of the test stimulus was one of the other four SFs. A single standard speed of 8.64 deg/s (corresponding to 0.33 rev/s = 2.09 rad/s of the radial grating at the middle radius of the ring) was tested.

#### Procedure

The task and the design was the same as in Experiment 2; each test SF was tested separately in a double-random-staircase run of 28 trials for each direction, and each run was repeated three times in a random order.

### Results

**Figure 6.**
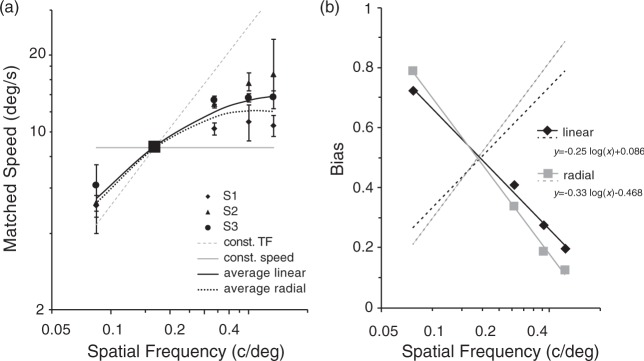
Results of Experiment 3. (a) Matched speed of the 0.15 c/deg stimulus is plotted as a function of the test spatial frequency. Symbols show individual results with 95% confidence intervals. The larger square shows the point of matching SF (not measured). The thick black curve denotes the fitted log-quadratic function to the averaged data. The fitted curve for the 4 c/rev condition in Figure 5(a) was adapted and superimposed as a dotted curve. (b) Estimated TF weighting, plotted as a function of spatial frequency. A linear function was fitted to the data (*r*^2 ^> .99). Corresponding data for the radial stimuli in Experiment 2 (standard speed of 0.33 rev/s) were adapted from Figure 5(b) and superimposed (gray squares and gray lines). The dashed lines represent bias for speed (1 − *b*).

Probit analysis (Finney, 1971; Johnson et al., 2013) was used to estimate the matched speed at 95% confidence intervals. Figure 6(a) shows the matched speed as a function of the test SF. Again, the PSE values fell between the area bounded by speed with constant TF and the veridical base speed of the test stimuli as shown in Figure 5(a), indicating overestimations and underestimations of perceived speed in higher and lower test SFs, respectively, when compared with the 0.15 c/deg matching stimulus.

Corresponding data from Experiment 2 were converted in the way as described in the Apparatus and Stimuli section and were plotted together in Figure 6(a). The pattern of results is very similar to that of the radial stimuli, as the fitted log-quadratic function (*r*^2 ^> .99) is comparable to the adapted curve from Figure 5(a). While the overestimation was somewhat larger for the linear gratings, this difference is surprisingly small, given many possible confounds such as imperfect matching of SFs, different stimulus windows, or larger SF artefacts at the stimulus edges of linear gratings, which are inevitable for the different kinds of stimuli. This result suggests that the spinner illusion is a consequence of general speed computation from spatiotemporal frequencies, rather than manifestation of some idiosyncratic rotation-specific effects.

Figure 6(b) depicts the TF weighting on perceived speeds of drifting gratings in the same way as in Figure 5(b) but as a function of linear SF. The data for the corresponding condition in Experiment 2 with radial gratings (standard speed of 0.33 rev/s) are adapted from Figure 5(b) and are plotted along in this figure. The decline of TF weighting is very similar for the two types of stimuli. The small difference in the slopes may be due to several factors as noted earlier.

We should also note that the shape of the curve is similar to that of speed matching by Jogan and Stocker (2015; their Figure 5(b), test stimuli of A–D), although they did not discuss this “single-channel” response in detail, because their focus was on integration of such responses in compound SF stimuli.

## General Discussion

The spinner illusion demonstrates that perceived speed is affected by the number of dots even when they move at a constant speed. We have confirmed that this effect generalizes to radial and linear forms of sinusoidal gratings. The spinner illusion therefore is considered to reflect a general speed overestimation for high SFs that has been reported in the literature. The effect of motion blur, if any, is not a necessary condition.

### The spinner illusion as a TF bias in spatiotemporal integration

As the spinner illusion occurs in a very similar way for radial and linear gratings, the effect is consistent with a number of studies that showed higher perceived speed for higher SFs with linear gratings (Brooks, Morris, & Thompson, 2011; Campbell & Maffei, 1981; Chen et al., 1998; Diener et al., 1976; McKee & Silverman, 1986). The asymptotic curve in our Figure 6(a) parallels with Figure 6 in McKee & Silverman (1986) and Figure 3 of Brooks et al. (2011). Smith and Edgar showed opposite results of speed underestimation for high SFs, but as they discussed (1990, p. 1473), their results are not necessarily in conflict because they used higher range of SFs (0.5–2 c/deg) than ours (0.08–0.68 c/deg) and Diener et al.’s (0.01–0.07 c/deg); the overall SF tuning might be inverted U-shaped as observed in Campbell and Maffei (1981).

Taken together, these results support a bias toward TF in the spinner illusion, as Anstis and Ho (2014) proposed. TF should increase along with increasing SF in order to keep the speed constant, and this higher TF could bias speed coding. Although we can make speed and TF judgments independently (Smith & Edgar, 1990, p. 1469), naïve judgments may be erroneously contaminated by the TF. This, however, does not seem to explain our compelling perception of speed difference in the spinner illusion demonstrations. Also, the results were consistent across naïve and trained participants in our experiments. The TF bias therefore could be an innate property in speed perception.

It is conceived that the visual system initially takes separate measures in space and time (i.e., SF and TF), and then integrates these measures into the metric of speed at the level of V1 complex cells to the area MT/V5 both in macaques (Priebe, Cassanello, & Lisberger, 2003; Priebe, Lisberger, & Movshon, 2006) and also in humans (Lingnau, Ashida, Wall, & Smith, 2009). Integration across space and time is then required for perceiving global pattern of linear or circular motion (e.g., Morrone, Burr, & Vaina, 1995). While the TF bias may arise at any of these stages, the results of similar spinner effects on circular and translating motions might suggest that it occurs before the stage of global integration.

The exact mechanism of the TF bias in speed perception is yet to be investigated, but our results of stronger TF bias for lower SFs might be explained by hypothetical receptive fields that are not large enough for the low-SF stimuli. The nominal SF of our stimuli extends down to 0.08 c/deg, that is, 12.5 deg/c. This is larger than the human population receptive field sizes, measured by using fMRI, in most visual areas including putative human MT and MST up to the eccentricity of 10° in the periphery (Amano, Wandell, & Dumoulin, 2009). Appendix Movie 4 shows a hypothetical receptive field that covers less than one cycle of a low-SF drifting grating, which is therefore seen as mostly flickering with very little motion signal. On the other hand, the same receptive field can register several moving bars of a high-SF grating, so the direction of motion is now unambiguous—and also looks fast. This simple model explains both the basic spinner effect, that fine bars appear to move faster than coarse bars, and also the fact that speed judgments of coarse moving bars are more strongly weighted by TF than by actual velocity.

Note that the illusion could be understood in accordance with the gradient-based models of motion detection (e.g., Anstis, 1967, 1990; Johnston, McOwan, & Buxton, 1992; Marr & Ullman, 1981). In the case of sinusoidal modulation, because *d*/*dt*[sin(*ft*)] = *f*cos(*ft*), maximum temporal gradient is proportional to TF. Speed perception could be understood as biased toward temporal gradient instead of TF.

### Ecological Interpretations

For periodic grating patterns, SF (or RSF) is a reciprocal of the size of each bar. The spinner effect therefore parallels the classic report of Brown (1931) that “other things being equal larger figures are phenomenally slower” (p. 222). It is, however, not fully understood why speed perception depends on size. A possible account could refer to speed constancy across distances; as an object comes closer, the retinal size and speed increase when the physical speed is constant. While Brown (1931) argued that constancy of velocity (i.e., speed) is not fully deducible from size constancy, Rock, Hill, and Fineman (1968) concluded that size constancy and speed constancy are indeed related, from the results of experiments without visible frames of references.

The rotating spinner illusion is not readily explained by speed constancy because RSF does not change across distances. On the other hand, the two-dimensional SF components along the *x-y* coordinates do change with distance for both linear or radial gratings, while TF remains constant. TF is therefore a more invariant measure than SF across distances, which may be one reason for more dependence on TF in speed computations.

Other possible explanations might refer to the statistical tendency that lower SFs are likely more stable than higher SFs in the scene. This is related to the hypothesis of Bayesian prior for slow motion (Jogan & Stocker, 2015; Vintch & Gardner, 2014; Weiss, Simoncelli, & Adelson, 2002), reflecting the fact that the world is mostly stable while smaller objects can move around, although this might not always hold (Hammett, Champion, Thompson, Morland, 2007).

### Limitations

There remains a question: Why does the speed appear to get faster steadily whenever the number of discs increase in the original spinner illusion, while the perceived speed saturated rapidly in our experiments? A potential cause for the discrepancy might be the high-SF harmonic components in the dot stimuli, as Brooks et al. (2011) showed that a complex grating of 1*f* + 2*f* SF components yielded less asymptotic curves of speed overestimation for higher SFs than a simple grating. Smith and Edgar (1991) also revealed nonlinear ways of combining element speeds in various complex gratings, but how this is related to the spinner illusion remains open for further investigation. Another factor could be the retinal blur due to sharp edges that might add to the effect, as Ho and Anstis (2013) originally postulated.

The physical contrast was always held constant at 50% in our experiments, and there could have been a confound of perceived contrast across SFs, since perceived speed depends on contrast (Thompson, 1982). We do not, however, consider that this effect is crucial in our case, because the effect of contrast is less clear for high-contrast stimuli. While Stone and Thompson (1992) showed that the effect does not saturate up to 70% contrast, Smith and Edgar (1990) informally noted that the perceived speed was independent of contrast above 10%.

Another remaining question is the generality of the spinner effect for second-order stimuli that cannot be computed from spatial and temporal frequencies, which is currently under investigation by one of the authors (A. H.).

## Supplementary Material

Supplementary material

Supplementary material

Supplementary material

Supplementary material

## Author Note

Preliminary results were presented at ECVP 2014 by H. A. and A. K.

## Supplemental Material

The online appendix movies are available at http://journals.sagepub.com/doi/suppl/10.1177/2041669517707972.

## Note

1 We use the unit of c/rev for RSF because it is more intuitive and directly comparable to the number of dots in the original spinner illusion. We also use the unit of rev/s for speed, for consistency. Corresponding values in SI units (c/rad and rad/s, respectively) are presented in parentheses in the methods sections.

## References

[bibr2-2041669517707972] Amano K. Wandell B. A. Dumoulin S. O. (2009) Visual field maps, population receptive field sizes, and visual field coverage in the human MT+ complex. Journal of Neurophysiology 102: 2704–2718. doi:10.1152/jn.00102.2009. 19587323 10.1152/jn.00102.2009 PMC2777836

[bibr3-2041669517707972] Anstis S. (1990) Motion aftereffects from a motionless stimulus. Perception 19: 301–306. 2267142 10.1068/p190301

[bibr4-2041669517707972] Anstis S. (1967) Visual adaptation to gradual change of intensity. Science 155: 710–712. 6016954 10.1126/science.155.3763.710

[bibr5-2041669517707972] Anstis S. Ho A. (2014) Apparent speed of a rotating disk varies with texture density. Journal of Vision 14: 1333, doi:10.1167/14.10.1333.

[bibr6-2041669517707972] Apthorp D. Schwarzkopf D. S. Kaul C. Bahrami B. Alais D. Rees G. (2013) Direct evidence for encoding of motion streaks in human visual cortex. Proceedings of the Royal Society of London. B: Biological Sciences 280: 20122339, doi:10.1098/rspb.2012.2339. 10.1098/rspb.2012.2339 PMC3574303 23222445

[bibr7-2041669517707972] Brainard D. H. (1997) The Psychophysics Toolbox. Spatial Vision 10: 433–436. 9176952

[bibr8-2041669517707972] Brooks K. R. Morris T. Thompson P. (2011) Contrast and stimulus complexity moderate the relationship between spatial frequency and perceived speed: Implications for MT models of speed perception. Journal of Vision 11: 19, doi:10.1167/11.14.19. 10.1167/11.14.19 22194317

[bibr9-2041669517707972] Brown J. F. (1931) The visual perception of velocity. Psychologische Forschung 14: 199–232. doi:10.1007/BF00403873.

[bibr10-2041669517707972] Campbell F. W. Maffei L. (1981) The influence of spatial frequency and contrast on the perception of moving patterns. Vision Research 21: 713–721. 7293002 10.1016/0042-6989(81)90080-8

[bibr11-2041669517707972] Campbell F. W. Robson J. G. (1968) Application of Fourier analysis to the visibility of gratings. The Journal of Physiology 197: 551–566. 5666169 10.1113/jphysiol.1968.sp008574 PMC1351748

[bibr12-2041669517707972] Cavanagh P. Tyler C. W. Favreau O. E. (1984) Perceived velocity of moving chromatic gratings. Journal of the Optical Society of America A 1: 893–899. 10.1364/josaa.1.000893 6470841

[bibr13-2041669517707972] Chen Y. Bedell H. E. Frishman L. J. (1998) The precision of velocity discrimination across spatial frequency. Percept Psychophys 60: 1329–1336. 9865074 10.3758/bf03207995

[bibr14-2041669517707972] Diener H. C. Wist E. R. Dichgans J. Brandt T. (1976) The spatial frequency effect on perceived velocity. Vision Research 16: 169–176. 1266057 10.1016/0042-6989(76)90094-8

[bibr15-2041669517707972] Finney D. J. (1971) Probit analysis, 3rd ed Cambridge, England: Cambridge University Press.

[bibr16-2041669517707972] Geisler W. S. (1999) Motion streaks provide a spatial code for motion direction. Nature 400: 65–69. doi:10.1038/21886. 10403249 10.1038/21886

[bibr17-2041669517707972] Hammett S. T. Champion R. A. Thompson P. G. Morland A. B. (2007) Perceptual distortions of speed at low luminance: Evidence inconsistent with a Bayesian account of speed encoding. Vision Research 47: 564–568. doi:10.1016/j.visres.2006.08.013. 17011014 10.1016/j.visres.2006.08.013

[bibr19-2041669517707972] Ho, A., & Anstis, S. (2013). *The Coyote Illusion: Motion blur increases apparent speed*. Paper presented at the Best illusion of the year contest 2013. Retrieved from http://illusionoftheyear.com/2013/05/the-coyote-illusion-motion-blur-increases-apparent-speed/.

[bibr20-2041669517707972] Jogan M. Stocker A. A. (2015) Signal integration in human visual speed perception. Journal of Neuroscience 35: 9381–9390. doi:10.1523/JNEUROSCI.4801-14.2015. 26109661 10.1523/JNEUROSCI.4801-14.2015 PMC6605192

[bibr21-2041669517707972] Johnson R. M. Dahlgren L. Siegfried B. D. Ellis M. D. (2013) Acaricide, fungicide and drug interactions in honey bees (Apis mellifera). PLoS One 8: e54092, doi:10.1371/journal.pone.0054092. 23382869 10.1371/journal.pone.0054092 PMC3558502

[bibr22-2041669517707972] Johnston A. McOwan P. W. Buxton H. (1992) A computational model of the analysis of some first-order and second-order motion patterns by simple and complex cells. Proceedings of the Royal Society of London. B: Biological Sciences 250: 297–306. doi:10.1098/rspb.1992.0162. 10.1098/rspb.1992.0162 1362996

[bibr23-2041669517707972] Lingnau A. Ashida H. Wall M. B. Smith A. T. (2009) Speed encoding in human visual cortex revealed by fMRI adaptation. Journal of Vision 9: 3.1–3.14. doi:10.1167/9.13.3. 10.1167/9.13.3 20055536

[bibr24-2041669517707972] Marr D. Ullman S. (1981) Directional selectivity and its use in early visual processing. Proceedings of the Royal Society of London. B: Biological Sciences 211: 151–180. 6111795 10.1098/rspb.1981.0001

[bibr25-2041669517707972] McKee S. P. Silverman G. H. Nakayama K. (1986) Precise velocity discrimination despite random variations in temporal frequency and contrast. Vision Research 26: 609–619. 3739236 10.1016/0042-6989(86)90009-x

[bibr26-2041669517707972] Morrone M. C. Burr D. C. Vaina L. M. (1995) Two stages of visual processing for radial and circular motion. Nature 376: 507–509. doi:10.1038/376507a0. 7637781 10.1038/376507a0

[bibr27-2041669517707972] Nishida S. (2011) Advancement of motion psychophysics: Review 2001–2010. Journal of Vision 11: 11, doi:10.1167/11.5.11. 10.1167/11.5.11 22144564

[bibr28-2041669517707972] Palmer S. (1999) Vision Science: From Photons to Phenomenology, Cambridge, MA: MIT Press.

[bibr29-2041669517707972] Peirce J. W. (2007) PsychoPy—Psychophysics software in Python. Journal of Neuroscience Methods 162: 8–13. doi:10.1016/j.jneumeth.2006.11.017. 17254636 10.1016/j.jneumeth.2006.11.017 PMC2018741

[bibr30-2041669517707972] Pelli D. G. (1997) The VideoToolbox software for visual psychophysics: transforming numbers into movies. Spatial Vision 10: 437–442. 9176953

[bibr31-2041669517707972] Priebe N. J. Cassanello C. R. Lisberger S. G. (2003) The neural representation of speed in macaque area MT/V5. Journal of Neuroscience 23: 5650–5661. 12843268 10.1523/JNEUROSCI.23-13-05650.2003 PMC2553808

[bibr32-2041669517707972] Priebe N. J. Lisberger S. G. Movshon J. A. (2006) Tuning for spatiotemporal frequency and speed in directionally selective neurons of macaque striate cortex. Journal of Neuroscience 26: 2941–2950. doi:10.1523/JNEUROSCI.3936-05.2006. 16540571 10.1523/JNEUROSCI.3936-05.2006 PMC2532672

[bibr33-2041669517707972] Rock I. Hill A. L. Fineman M. (1968) Speed constancy as a function of size constancy. Perception & Psychophysics 4: 37–40.

[bibr34-2041669517707972] Sachs M. B. Nachmias J. Robson J. G. (1971) Spatial-frequency channels in human vision. Journal of the Optical Society of America 61: 1176–1186. 5121888 10.1364/josa.61.001176

[bibr35-2041669517707972] Smith A. T. Edgar G. K. (1990) The influence of spatial frequency on perceived temporal frequency and perceived speed. Vision Research 30: 1467–1474. 2247956 10.1016/0042-6989(90)90027-i

[bibr36-2041669517707972] Smith A. T. Edgar G. K. (1991) Perceived speed and direction of complex gratings and plaids. Journal of the Optical Society of America A 8: 1161–1171. 10.1364/josaa.8.001161 1886008

[bibr37-2041669517707972] Stone L. S. Thompson P. (1992) Human speed perception is contrast dependent. Vision Research 32: 1535–1549. 1455726 10.1016/0042-6989(92)90209-2

[bibr38-2041669517707972] Thompson P. Brooks K. Hammett S. T. (2006) Speed can go up as well as down at low contrast: implications for models of motion perception. Vision Research 46: 782–786. doi:10.1016/j.visres.2005.08.005. 16171842 10.1016/j.visres.2005.08.005

[bibr39-2041669517707972] Thompson P. G. (1982) Perceived rate of movement depends on contrast. Vision Research 22: 377–380. 7090191 10.1016/0042-6989(82)90153-5

[bibr40-2041669517707972] Vintch B. Gardner J. L. (2014) Cortical correlates of human motion perception biases. Journal of Neuroscience 34: 2592–2604. doi:10.1523/JNEUROSCI.2809-13.2014. 24523549 10.1523/JNEUROSCI.2809-13.2014 PMC6802749

[bibr41-2041669517707972] Weiss Y. Simoncelli E. P. Adelson E. H. (2002) Motion illusions as optimal percepts. Nature Neuroscience 5: 598–604. doi:10.1038/nn858. 12021763 10.1038/nn0602-858

